# Fabrication and efficacy evaluation of scopolamine-loaded dissolving microneedle for rapid antidepressant therapy

**DOI:** 10.1016/j.ijpx.2026.100601

**Published:** 2026-07-06

**Authors:** Yanping Wang, Yuwei Shi, Qiuyue Chen, Minghui Hu, Yue Zhao, Wanqing Ren, Kun Liu, Fangyi Hou, Yaru Cui, Yuning Ma, Mengzhen Xing, Xiwen Geng

**Affiliations:** a Experimental Center, Shandong University of Traditional Chinese Medicine, Jinan 250355, PR China; b Key Laboratory of Traditional Chinese Medicine Classical Theory, Ministry of Education, Shandong University of Traditional Chinese Medicine, Jinan 250355, PR China; c Innovative Institute of Chinese Medicine and Pharmacy, Shandong University of Traditional Chinese Medicine, Jinan 250355, PR China; d Research Institute of Marine Traditional Chinese Medicine, Shandong University of Traditional Chinese Medicine, Qingdao 266112, PR China; e Institute of Pharmacy (Institute of TCM Health Industrial Technolog, Shandong University of Traditional Chinese Medicine, Jinan 250355, PR China; f School of Chinese Pharmacy, Shanghai University of Traditional Chinese Medicine, Shanghai 201203, PR China

**Keywords:** Dissolving microneedles, Scopolamine, Rapid-acting antidepressant, Synaptic plasticity, BDNF/mTOR pathway, 1. Scopolamine hydrobromide (CAS: 6533-68-2), Shanghai Macklin Biochemical, China, 2. Sodium hyaluronate (CAS: 9067-32-7), Guangzhou Zhongguang Biotech, China, 3. Polyvinyl alcohol (CAS: 9002-89-5), Jiangxi Alpha High-Tech Pharm, China, 4. Dextran (CAS: 9004-54-0), Jinan Chemical Co., Ltd., China, 5. Acridine orange (CAS: 10127-02-3), Yuanye Biotech, China, 6. Polydimethylsiloxane microneedle mold (CAS: 63148-62-9), Beijing CAS Microneedle Tech, China

## Abstract

Depression is a global mental health crisis, and the rapid-acting antidepressant scopolamine (Scop) is limited by systemic administration drawbacks. This study developed a scopolamine-loaded bilayer dissolving microneedle (Scop-DMN) system for transdermal delivery and evaluated its antidepressant efficacy in a chronic restraint stress (CRS)-induced mouse model. Bilayer DMNs were fabricated via sequential centrifugal molding, with a polyvinyl alcohol (PVA) backing layer and a dextran‑sodium hyaluronate drug-loaded tip matrix. Physicochemical properties, biocompatibility, and in vivo therapeutic effects were systematically characterized. Scop-DMNs showed uniform conical morphology, high drug loading homogeneity, and complete dissolution within 2 min in porcine skin. In C57BL/6 J mice, Scop-DMNs significantly ameliorated CRS-induced depressive-like behaviors in open field, sucrose splash tests, and tail suspension tests 24 h post-administration, outperforming conventional intraperitoneal scopolamine. Mechanistically, Scop-DMNs robustly activated the brain-derived neurotrophic factor / mammalian target of rapamycin (BDNF/mTOR) pathway and upregulated synaptic proteins (postsynaptic density protein 95 (PSD95), Synapsin I (SynI)) in the medial prefrontal cortex (mPFC), reversing stress-induced neurotrophic and synaptic deficits. This Scop-DMN system provides a safe, minimally invasive, and effective transdermal platform for scopolamine delivery, offering a promising clinical strategy for rapid management of acute depressive episodes.

## Highlights

• A bilayer dissolving microneedle (DMN) system was developed for transdermal scopolamine delivery.
• Scop-DMNs dissolved rapidly in skin and showed superior antidepressant efficacy over intraperitoneal injection.
• Scop-DMNs activated the BDNF/mTOR pathway and reversed synaptic deficits in depressed mice.
• The microneedle platform offers a promising strategy for rapid antidepressant therapy with scopolamine.

## Graphical abstract

**Figure:**
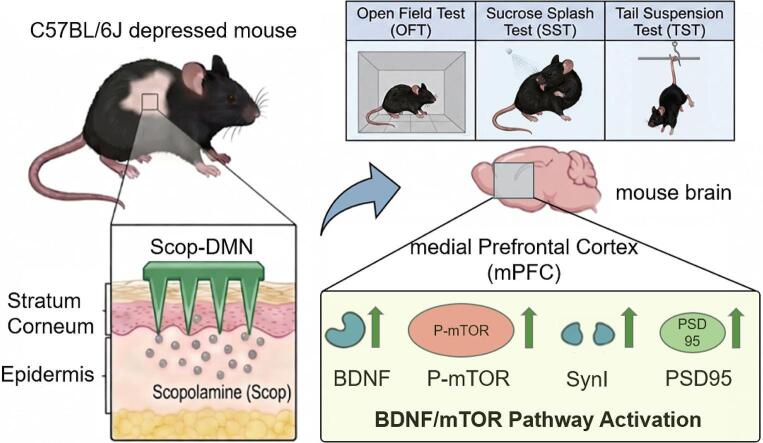

## 1 Introduction

Depression is a highly prevalent and disabling neuropsychiatric disorder, clinically characterized by persistent low mood, psychomotor retardation, and reduced motivation and goal-directed behavior (Li et al., 2025; Zhou et al., 2025). Epidemiological evidence indicates a substantial and growing global disease burden, marked by high incidence, frequent relapse, and persistently low remission rates (Arias-de la Torre et al., 2021; Brivio et al., 2023). Although monoaminergic antidepressants remain first-line therapies, their clinical utility is often limited by systemic adverse effects, tolerance/dependence liability, and clinically relevant discontinuation syndromes (Pillinger et al., 2025).

Accumulating evidence indicates that depression-related functional impairments are closely associated with synaptic dysfunction within the medial Prefrontal Cortex (mPFC)—a critical neural hub for emotional regulation and executive function. At the molecular level, this is characterized by compromised synaptogenesis and a deteriorated synaptic microenvironment driven by suppressed Brain-derived Neurotrophic Factor (BDNF) signaling and downstream hypoactivation of the mammalian Target of Rapamycin (mTOR) pathway. This cascade results in the downregulation of key synaptic proteins essential for structural remodeling and neurotransmission, such as Postsynaptic Density protein 95 (PSD95) and Synapsin I (SynI) (Parekh et al., 2022). Consequently, reversing synaptic failure via the BDNF/mTOR pathway has emerged as a convergent endpoint for rapid-acting antidepressant interventions (Parekh et al., 2022; Ren et al., 2025).

In the 1970s, Janowsky and colleagues proposed the cholinergic hypothesis of depression, suggesting that cholinergic hyperactivity—particularly excessive muscarinic receptor signaling-contributes importantly to depressive symptomatology (Dulawa and Janowsky, 2019). Scopolamine (Scop), a prototypical non-selective muscarinic antagonist, primarily blocks postsynaptic muscarinic receptors and thereby suppresses cholinergic transmission (Dulawa and Janowsky, 2019). Scop is a highly anticholinergic tropane alkaloid characterized by a low molecular weight (303.35 g/mol) and moderate lipophilicity. These physicochemical properties enable the molecule to efficiently permeate the blood-brain barrier (BBB) and exert direct pharmacological effects on the central nervous system (CNS). Clinically, scopolamine is widely used for perioperative sedation and adjunctive anesthesia, prophylaxis of motion sickness, and management of gastrointestinal spasm (Atif et al., 2022; Navarria et al., 2015). Although scopolamine transdermal patches are used clinically to prevent motion sickness, their onset of action is too slow to be a rapid choice, and they are not intended for the treatment of depression. Beyond these approved indications, accumulating experimental (preclinical) evidence demonstrates that scopolamine elicits rapid-onset (≈24–72 h) and sustained antidepressant-like effects in rodent models, supporting its potential as a mechanistically distinct alternative to conventional monoaminergic strategies (Yana et al., 2025; Wohleb et al., 2016). However, translation of these findings toward antidepressant applications has been impeded largely by administration-related constraints and a narrow therapeutic window: oral dosing shows poor and variable bioavailability due to extensive first-pass metabolism, whereas parenteral delivery requires medical supervision and is frequently associated with dose-limiting central anticholinergic adverse effects, including sedation, confusion, and memory impairment (Gao et al., 2024; Renner et al., 2005). These limitations highlight the need for an improved, minimally invasive delivery strategy that enables more controllable administration and better tolerability to advance scopolamine-based interventions for depression. If a new formulation method for scopolamine to exert a rapid antidepressant effect can be found, which is convenient to operate, safe and has a therapeutic effect comparable to the traditional administration method, the value of scopolamine in exerting a rapid antidepressant effect can be maximized.

Microneedle (MN) technology provides a minimally invasive and essentially painless approach to overcome the stratum corneum barrier, enabling reproducible drug deposition into the viable epidermis and upper dermis (Abdul Aziz et al., 2025). As a transdermal platform, MN delivery is commonly categorized into solid MN, coated MN, hollow MN, dissolving MN, and swelling MN, each offering distinct strengths in formulation flexibility, drug-loading capacity, and controlled release behavior (Pastore et al., 2015). Among them, dissolving microneedle (DMN) fabricated from water-soluble polymers have attracted particular interest because they rapidly dissolve within minutes, deliver drug without generating sharp waste, and are generally associated with good biocompatibility(Cong et al., 2024; Moawad et al., 2025; Nainggolan et al., 2024; Sartawi et al., 2022; Vora et al., 2023). In addition, depression is a chronic and recurrent disorder that ofter requires long-term and consistent medication adherence for optimal therapeutic outcomes. DMN enable convenient self-administration without the need for professional assistance, thereby improving patient compliance in daily management. The novel formulation of scopolamine-loaded microneedles can be prepared. It can then be explored whether this new formulation exerts a rapid antidepressant effect and whether this effect is similar to that of intraperitoneal injection of scopolamine for the treatment of depression. This approach could address the various drawbacks associated with the rapid antidepressant effect of scopolamine and provide a new administration method for scopolamine to achieve a rapid antidepressant effect.

Herein, we developed scopolamine-loaded dissolving microneedle (Scop-DMN) as a transdermal delivery platform. The formulation was systematically characterized in terms of the skin insertion capability, transdermal delivery efficiency, and biosafety of Scop-DMN. To examine its antidepressant potential in vivo, we employed a 14-day chronic restraint stress (CRS) paradigm to establish a depression-like phenotype in mice, followed by behavioral assessments to quantify stress-induced affective and motivational deficits. In parallel, medial prefrontal cortex (mPFC) tissue was collected for mechanistic interrogation of key synaptic plasticity-related pathways, focusing on BDNF, P-mTOR, PSD95, and SynI. Through integrating formulation development with behavioral phenotyping and molecular readouts, this study aims to determine whether transdermal delivery of Scop via DMN system can alleviate depression-like behaviors and normalize associated neurobiological alterations, thereby providing a practical alternative dosing strategy and a translational framework for expanding scopolamine-based interventions for major depressive disorder.

## 2 Materials and methods

### 2.1 Materials and animals

Scopolamine hydrobromide (SH, purity 98%) was purchased from Shanghai Macklin Biochemical Technology Co., Ltd. (Shanghai, China). Sodium hyaluronate (HA, Mw ≈ 10 kDa) was obtained from Guangzhou Zhongguang Biotechnology Co., Ltd. (Guangzhou, China). Polyvinyl alcohol (PVA; Mw 85–124 kDa) was purchased from Jiangxi Alpha High-Tech Pharmaceutical Co., Ltd. (Jiangxi, China). Dextran (Mw 40–60 kDa) was purchased from Jinan Chemical Co.,Ltd. (Jinan, China). Polydimethylsiloxane (PDMS) microneedle molds were acquired from Beijing CAS Microneedle Technology Ltd. (Beijing, China). The molds were designed to fabricate microneedle patches with a total area of 0.56 cm^2^, containing an array of 144 needles. The specific geometrical dimensions of the individual microneedle templates included a height of 500 μm, a base diameter of 300 μm, and an inter-needle spacing of 500 μm. The acridine orange/ethidium bromide (AO/EB) dual fluorescence staining kit was provided by Yuanye Biotech Co., Ltd. (Shanghai, China). All other chemicals were of reagent grade and obtained from commercial sources. Ex vivo porcine cadaver skin was obtained from Kaikai Tech Co., Ltd. (Shanghai, China). Embryonic mouse fibroblast (NIH/3 T3) cells were kindly provided by Lecturer Xin Zhang, Shandong University of Traditional Chinese Medicine (Shandong, China).

Male C57BL/6 J mice (8 weeks old, provided from the Laboratory Animal Center of Shandong University of Traditional Chinese Medicine) were used in this study. All mice were housed in a standard animal facility with a 12 h light/dark cycle (light from 20:00 to 08:00) with food and water ad libitum. The environment was maintained at 55 ± 2% relative humidity and a temperature of 21.0 ± 2.0 °C. All animal experiments were conducted according to the guidelines for laboratory animal care and use established by the National Institutes of Health (NIH), and were approved by the Experimental Animal Ethics Committee of Shandong University of Traditional Chinese Medicine (Approval No. SDUTCM-IACUC-20240508014).

### 2.2 Fabrication of Scop-DMN patches

To achieve rapid intradermal drug delivery, we fabricated Scop-dDMN with drug-loaded tips using a two-step injection molding method. Briefly, a 40% (*w/v*) polyvinyl alcohol (PVA) solution was prepared as the DMN backing solution. Meanwhile, a needle-tip solution containing 1% (*w/v*) dextran, 1% (*w/v*) sodium hyaluronate, and 0.044% (*w/v*) Scop was prepared. The low polymer concentration facilitates the preferential accumulation of scopolamine at the needle apex. This localized density is critical for maximizing drug delivery efficiency upon skin penetration.The high concentration and resulting high viscosity of the PVA backing serve as a diffusion barrier. This prevents the migration of the therapeutic agent from the tips into the backing during the drying phase, maintaining high drug density at the penetration site(Xuan et al., 2025). During the microneedle fabrication process, a needle-tip solution was injected into the cavities of a polydimethylsiloxane (PDMS) mold. A vacuum was subsequently applied for 30 min to ensure that the needle cavities were completely filled under negative pressure conditions. After the tips had sufficiently dried and formed, the PVA backing solution was added onto the mold and allowed to dry naturally at room temperature for 12 h, yielding well-structured Scop-DMN patches. Moreover, to elucidate the layered effect of the DMN fabricated using this process, we incorporated sodium fluorescein and Rhodamine B into the needle-tip solution and backing solution, respectively. The dual-fluorescence-labeled DMN was prepared in accordance with the aforementioned method. The drug loading efficiency at the MN tip (%) was calculated using Eq. (1), yielding a value of 2.15%.

(1) $$\text{Drug loading efficiency}\;\mathrm{at}\;\text{the}\;\mathrm{MN}\;\mathrm{tip}\;\left(\%\right)=\frac{M_{\text{Scop}}}{M_{\text{Scop}}+M_{\text{Dextran}}+M_{\text{Sodium hyaluronate}}}$$

### 2.3 Development and validation of HPLC method for Scop quantification

A high-performance liquid chromatography (HPLC, e2695, Waters, Milford, MA, USA) method was developed for the quantitative determination of Scop, utilized for the analysis of drug loading within DMN patches. A C18 column (250 mm × 4.6 mm, 5 μm, YMC Triart, Japan) was used with a mobile phase consisting of (A) an aqueous phase containing 0.25% sodium dodecyl sulfate (pH adjusted to 2.5 with phosphoric acid) and (B) acetonitrile in a ratio of 55:45 (*v/v*). The flow rate was 1.0 mL/min, the injection volume was 20 μL, the detection wavelength was 210 nm, and the column temperature was 30 °C. In accordance with the provisions of the 2025 Chinese Pharmacopoeia, the HPLC method was validated for specificity, linearity, sensitivity, precision, and accuracy.

### 2.4 Morphology, puncture resistance, solubility, and mechanical strength of Scop-DMN patches

#### 2.4.1 Morphological characterization

The morphology and structural integrity of Scop-DMN patches were observed using a 3D digital microscope (DSX1000, Olympus Corporation, Tokyo, Japan) and a scanning electron microscope (MIRA LMS, Tescan Brno, Czech Republic) at magnifications of ×50 to ×200 to evaluate needle height uniformity and tip sharpness. Prior to SEM observation, the samples were mounted on aluminum stubs using carbon conductive tape and sputter-coated with a thin layer of gold. The SEM images were acquired at an accelerating voltage of 15 kV. Furthermore, an inverted fluorescence microscope (SOPTOP ICX41, Ningbo Sunny Instruments Ningbo, China) was employed to image the rhodamine B-labeled base layer and the sodium fluorescein-loaded needle-tips, thereby assessing the stratification effect of the DMN.

#### 2.4.2 Skin insertion and mechanical strength

The skin insertion ability of Scop-DMN was evaluated through an ex vivo porcine skin insertion study. After pressing the DMN against the stratum corneum side of the skin for 20 s, the skin was stained with 4 mg/mL trypan blue solution, and penetration performance was assessed by observing the stained micropores array on the skin surface and calculating the percentage of complete skin insertion, according to Eq. (2).

(2) $$\text{Percentage of complete skin insertion}\;\left(\%\right)=\frac{\text{Number of stained micropores}}{\text{Number of}\;\mathrm{all}\;\text{micropores}\;\mathrm{on}\;\mathrm{MN}\;\text{molding}}$$

The penetrated skin was then placed in 4% paraformaldehyde, embedded in parafffn, sectioned, stained with hematoxylin and eosin (H&E), and the intradermal insertion depth of Scop-DMN was examined using a fuorescence microscope (ICX41, Soptop, Ningbo, China). Meanwhile, the mechanical strength of DMN was evaluated using a texture analyzer (TA.XTC20,Baosheng, Shanghai, China) with the needle tips oriented upwards on a horizontal stage, a probe compression force of 0.049 N, a movement speed of 1.0 mm/s, and a downward displacement of 0.5 mm was applied. The force-displacement curve between the force exerted on the DMN and the tip displacement was recorded and plotted.

#### 2.4.3 Intradermal dissolution performance

The dissolution behavior of Scop-DMN was evaluated using an ex vivo porcine skin model. The DMN patch was placed on the stratum corneum of the skin and pressed with a force of 20 N/cm^2^ (home-made applicator) for 20 s. This pressure was selected to ensure uniform penetration of the needle array through the epidermal barrier. Following the initial 20-s compression to facilitate stable insertion and initiate tip dissolution. The force was removed (H. Liu et al., 2023), and the patch remained adhered to the skin surface for 2 min to allow for the hydration of the polymeric tips. Subsequently, the DMN patch was peeled off, and the height of the residual needles was measured under a stereomicroscope.

### 2.5 Transdermal delivery efficiency of Scop-DMN

We simultaneously prepared Scop-DMN and Scop-patch (without needles) with identical drug loadings, and evaluated the transdermal delivery efficiency of both formulations using C57BL/6 J mice. The mice were anesthetized via isoflurane inhalation, followed by abdominal hair removal using clippers and a depilatory cream. Twenty-four hours after hair removal, Scop-DMN or Scop-patch (without needles) was applied to the depilated skin areas of the mice (one unit per mouse). The DMN or patch was pressed against the skin for 20 s using an home-made applicator (20 N/cm^2^), and was then secured with medical tape. At 6, 12, and 24 h post-administration, three mice were randomly selected from each treatment group, the residual drug amounts remaining in the DMN or patch on the skin surface was extracted and quantified via HPLC to determine the transdermal drug delivery efficacy of each formulation.

### 2.6 Biological safety studies

#### 2.6.1 In vitro live/dead cell staining

The biocompatibility of Scop-DMN was assessed via live/dead staining. Microneedle extracts were prepared by immersing one DMN array in DMEM (1 mL) for 24 h at 37 °C, followed by filtration through a 0.22 μm membrane. Pure DMEM and DMEM containing 5% DMSO served as negative and positive controls, respectively. NIH/3 T3 cells seeded in confocal dishes (2 × 10^4^ cells/dish) were cultured for 24 h, then exposed to the extracts for an additional 24 h. Live/dead staining was performed using acridine orange/ethidium bromide (AO/EB) for 20 min, and images were captured using a fluorescence microscope (ICX41, SOPTOP, China).

#### 2.6.2 Erythrocyte hemolysis rate

For the hemolysis assay, fresh anticoagulated blood from Sprague-Dawley rats was centrifuged (1000 rpm, 10 min, 4 °C) to collect erythrocytes, which were washed with Tris buffer until the supernatant was clear and then diluted to 5% (*v*/v) with Tris buffer. One Scop-DMN patch was dissolved in 1 mL of normal saline to obtain the test sample. Tris buffer with 0.1% Triton X-100 and normal saline served as positive and negative controls, respectively. Erythrocyte suspension (20 μL) was mixed with 1 mL of each sample and incubated at 37 °C with shaking (200 rpm) for 1 h. After centrifugation (10,000 rpm), the supernatant was photographed and its absorbance measured at 540 nm using a microplate reader (Multiskan FC, Thermo Fisher Scientific, USA). The hemolysis rate was calculated using the following Eq. (3):

(3) $$\text{Hemolysis rate}\;\left(\%\right)=\frac{\left(H-H_{n}\right)}{\left(H_{p}-H_{n}\right)}\times 100\%$$

where H, Hn, and Hp represent the absorbance of the Scop-DMN sample, negative control, and positive control, respectively.

#### 2.6.3 In vivo skin irritation test

The skin irritation of Scop-DMN was evaluated using three C57BL/6 J mice. The mice were anesthetized via isoflurane inhalation, followed by abdominal hair removal using clippers and a depilatory cream. The Scop-DMN patches were applied to the upper-left abdominal quadrant for 12 h. Subsequently, all patches were removed, and the occurrence of skin irritation reactions, such as erythema and edema, was observed and recorded at 0, 6, 12, and 24 h post-removal.

### 2.7 Construction of a mouse model of depression

After a 7-day acclimatization period, forty-eight C57BL/6 J mice were stratified by baseline body weight and randomly assigned (computer-generated random number sequence) to four groups (*n* = 12/group): (1) Control + blank microneedle patch (Control); (2) chronic restraint stress (CRS) + blank microneedle patch (CRS); (3) CRS + scopolamine hydrobromide via intraperitoneal injection (Scop-i.p); and (4) CRS + scopolamine-loaded bilayer dissolving microneedle patch (Scop-DMN). Group allocation was completed prior to CRS induction to ensure balanced distribution of baseline physiological parameters. All interventions were administered at equivalent scopolamine doses, with vehicle controls matched for volume and administration route (Drevets et al., 2013).

To evaluate the rapid antidepressant efficacy of scopolamine, a systemic target dose of 25 μg/kg was established based on prior literature(Yu et al., 2018). Given that the cohort of mice exhibited an average body weight of 30.0 g, the required absolute therapeutic mass of scopolamine per animal was determined to be approximately 750.0 ng. Therefore, in the subsequent treatment, the dosage of scopolamine loaded on the microneedle preparation used for each mouse was 750.0 ng.

For the comparison group involving conventional systemic administration, an intraperitoneal (i.p.) injection protocol was rigidly cross-calibrated. Scopolamine hydrobromide was dissolved in sterile 0.9% NaCl solution. The dosage of scopolamine in the intraperitoneal injection control group was still 25 μg/kg, dissolved in 0.9% physiological saline, with a volume of 10 mL per kg of mouse body weight. This dual calibration method minimized the dosage differences and ensured that the absolute amount of scopolamine introduced through the transdermal microneedle array and the traditional intraperitoneal injection route was exactly the same.

CRS was induced as previously established (Seo et al., 2017), with minor modifications to improve reproducibility and welfare compliance. Briefly, mice were restrained individually in well-ventilated polypropylene tubes (50 mL centrifuge tubes with 8–10 evenly distributed 2-mm-diameter ventilation holes) positioned horizontally (Yun et al., 2010). Animals were gently introduced head-first into the tube, and the open end was secured with a perforated rubber stopper to prevent escape while maintaining airflow. Restraint was applied for 6 h/day (09:00–15:00) for 14 consecutive days. Control mice were handled in parallel but remained in their home cages; to minimize metabolic and circadian confounders, they underwent concurrent 6-h daily fasting and water deprivation (09:00–15:00). On day 13, dorsal hair was removed using electric clippers followed by depilatory cream (Nair™, Church & Dwight Co., Inc.) to expose a 1.5 × 1.5 cm^2^ area for microneedle application. On day 14, immediately after the final CRS session, interventions were administered as follows: Control and CRS groups received intraperitoneal (i.p.) sterile 0.9% NaCl (10 mL/kg); the Scop-i.p group received i.p. scopolamine hydrobromide (25 μg/kg, dissolved in 0.9% NaCl); and the Scop-DMN group received topical application of scopolamine-loaded bilayer dissolving microneedle patches delivering an equivalent scopolamine dose (25 μg/kg) under standardized pressure (20 N/cm^2^for 20 s) using a custom applicator. Specifically, the packaging of the DMN patch was opened, and one side of the needle tip was attached to the exposed part of the mouse's skin. After 20 s of press, the needle tip was allowed to enter the skin and waited for 2 min, in order to ensure complete drug delivery before being removed.

### 2.8 Behavioral test

Behavioral performance was assessed using the Open Field Test (OFT), Sucrose Splash Test (SST), and Tail Suspension Test (TST). All behavioral assays were performed in three independent cohorts to ensure reproducibility. Only one behavioral test was conducted per day for each mouse, and all procedures were carried out during the dark phase. Prior to testing, mice were habituated to the experimental room for acclimation. All experiments were conducted in a quiet, temperature-controlled environment under dim red light to maintain consistent conditions and minimize external disturbance.

#### 2.8.1 Open field test (OFT)

The dimensions of the open field box were 50 cm (length) × 50 cm (width) × 50 cm (height) with a camera on the top (sampling rate: 15 frames /s). The test period for each mouse was 6 min. The behavior of the mice was monitored and captured using an infrared camera system in conjunction with a video synthesizer. The mice’ s movement trajec­tories were recorded by the XR-Super Maze video tracking and analysis system, and the following behavioral parameters were analyzed: total distance, distance in the central area, number of times entered into the central area, and duration in the central area.

#### 2.8.2 Sucrose splash test (SST)

The sucrose splash test (SST) was used to assess motivational/self-care–related grooming behavior, which is sensitive to depressive-like states. SST was performed 24 h after the final intervention. Each mouse was individually placed in a clean, empty cage, and 10% (*w*/*v*) sucrose solution was sprayed onto the dorsal coat near the base of the tail using a hand-held atomizer (2–3 uniform sprays from a constant distance). Behavior was video-recorded for 6 min using a top-mounted camera under the same testing conditions as other behavioral assays. Grooming was defined as stereotyped self-cleaning behaviors including licking of the fur and forepaws, face washing, and scratching. The following parameters were quantified: latency to initiate grooming (s) and total grooming duration (s) during the recording period. After each trial, the cage was replaced/cleaned to eliminate olfactory cues. Scoring was performed by an experimenter blinded to group allocation.

#### 2.8.3 Tail suspension test (TST)

The tail suspension test (TST) was performed to evaluate depressive-like behavior as previously described (Seo et al., 2012). Testing was conducted 24 h after the final intervention under standardized environmental conditions (23 ± 1 °C; 55 ± 5% relative humidity; ambient noise < 45 dB). Each mouse was suspended by the tail using adhesive tape affixed approximately 1 cm from the tail tip and attached to a horizontal bar positioned 50 cm above the surface. Sessions were recorded for 6 min using a ceiling-mounted camera. Immobility was defined as the absence of escape-directed movements, excluding minor movements required for respiration or postural adjustment. Total immobility time (s) was quantified over the entire 6-min session. All apparatus surfaces were cleaned with 75% (*v*/v) ethanol between animals. Behavioral scoring and analysis were conducted by investigators blinded to treatment groups.

### 2.9 Tissue extraction

Following the behavioral tests, the mice in each group were subjected to a 12 h fast and subsequently anesthetized via intraperitoneal administration of 2% sodium pentobarbital at a rate of 0.1 mL per 10 g of mice. The brain was severed and stripped with a guillotine, and the intact brain tissue was removed and placed on a petri dish on ice. The tissue of the medial prefrontal cortex (mPFC) was removed according to the brain atlas, and placed in a grinding tube for freezing in liquid nitrogen and stored at −80 °C.

### 2.10 Western blot detection

Mouse brain tissues were homogenized in lysis buffer (Beyotime Biotechnology Co., Ltd., Shanghai, China; Cat. No. P0013B) supplemented with protease inhibitors (PMSF, Beyotime Biotechnology Co., Ltd., Shanghai, China; Cat. No. ST506) for 30 min, followed by centrifugation at 13,000 rpm for 15 min at 4 °C. The BCA Protein Quantification Kit (Beyotime Biotechnology Co., Ltd., Shanghai, China; Cat. No. P0010S) instructions was used to determine the protein concentration of the samples, and then the proteins were denaturated at 95 °C for 10 min. Protein samples (30-50 μg) were resolved on 10% SDS-PAGE gels and subsequently transferred to polyvinylidene difluoride (PVDF) membranes (Millipore Corporation, Billerica, MA, USA; ISEQ00010) using an ice-cold transfer buffer (Wuhan Servicebio Technology Co., Ltd., Wuhan, Hubei, China; Cat. No. G2028) via electrotransfer for 1 h. The membranes were first blocked using a rapid blocking solution (Beyotime Biotechnology Co., Ltd., Shanghai, China; Cat. No. P0252) and subsequently incubated with the specified primary antibodies overnight at 4 °C: phospho-mTOR (Ser2448) (1:1000; Proteintech, Cat. No. 80596-1-RR), PSD95 (1:1000; Proteintech, Cat. No. 20665-1-AP), Synapsin I (1:1000; Proteintech, Cat. No. A6442), BDNF (1:1000; Proteintech, Cat. No. 28205-1-AP), and GAPDH (1:1000; Proteintech, Cat. No. 60004-1-Ig). After three 10-min washes in TBST (Wuhan Servicebio Technology Co., Ltd., Cat. No. G0001), membranes were incubated for 2 h at room temperature with HRP-conjugated secondary antibodies: goat anti-rabbit IgG (H + L) (1:10,000; PTG, Cat. No. RGAR001) and goat anti-mouse IgG (H + L) (1:10,000; PTG, Cat. No. RGAM001). Immunoreactive bands were visualized using enhanced chemiluminescence (ECL) reagent (Thermo Fisher Scientific, Cat. No. 34080) and captured with a ChemiDoc MP Imaging System (Bio-Rad). Band intensities were quantified using ImageJ software (NIH, v1.54f); target protein levels were normalized to GAPDH loading control and expressed as fold-change relative to the Control group (mean ± SD, *n* = 3 per group).

### 2.11 Statistical analysis

Using Graph Pad Prism 9.5.0 software, the Kolmogorov-Smirnov test and Levene’ s test were initially used to assess the normality of distri­bution and homogeneity of variance, respectively. For comparisons involving multiple groups, one-way ANOVA followed by the Tukey post hoc test or two-way ANOVA followed by the Bonferroni post hoc test was employed. Data are presented as mean ± standard error of the mean (SEM). A *p*-value of less than 0.05 was considered to indicate statistical significance.

## 3 Results

### 3.1 Morphology, puncture resistance, solubility, mechanical strength, and delivery of Scop-DMN patches

**Fig. 1:**
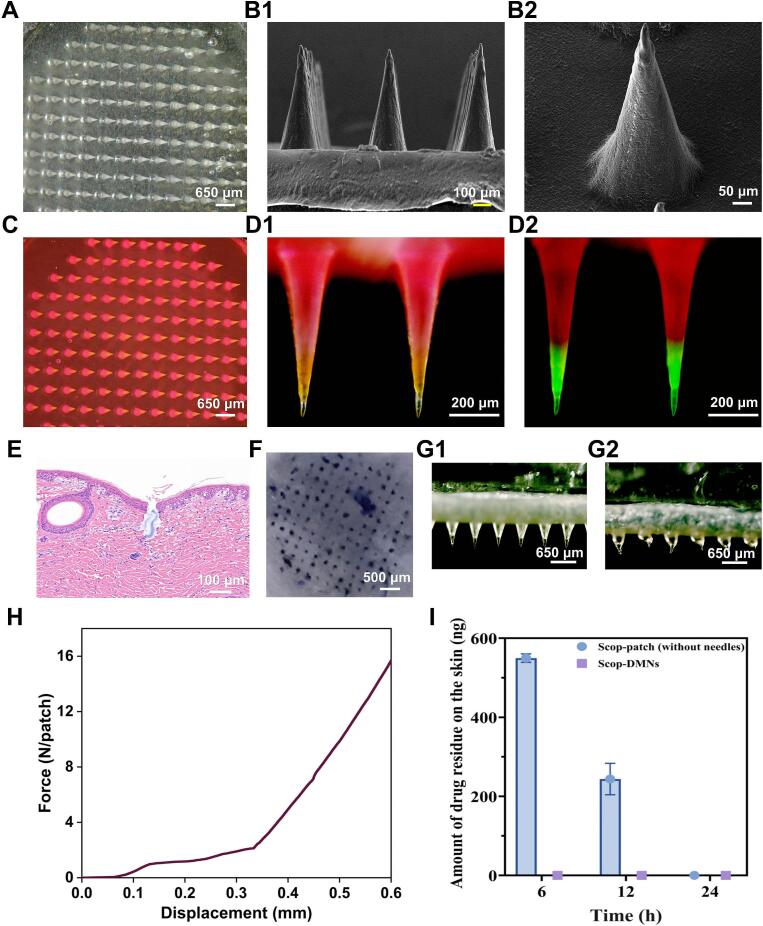
Characterization and evaluation of Scop-DMN patches. (A) 3D digital microscope image of the DMN array, scale bar = 650 μm. (B1-B2) SEM images, scale bars = 100 μm and 50 μm, respectively. (C) 3D digital microscope image of the DMN array fabricated with sodium fluorescein (tip layer) and rhodamine B (backing layer), scale bar = 650 μm. (D1-D2) Fluorescence microscopy images showing layer compartmentalization using sodium fluorescein (tip layer) and rhodamine B (backing layer), scale bars = 200 μm. (E) H&E staining of mouse dorsal skin after insertion demonstrating microchannel formation, scale bar = 100 μm. (F) Trypan blue staining of porcine skin confirming effective microporation, scale bar = 500 μm. (G1-G2) In vitro dissolution images of scopolamine-loaded DMNs: (G1) DMNs immediately after insertion into porcine skin (0 min), scale bar = 650 μm; (G2) DMNs 1 min after insertion into porcine skin, scale bar = 650 μm. (H) Force-displacement curve of the microneedles. (I) Amounts of Scop residue on the skin surface after application with Scop-DMN and Scop-patch (without needles) on live mice for 24 h, respectively (*n* = 3).

As shown in Figs. 1A, 3D digital microscopy demonstrated that the microneedle arrays were uniformly arranged with well-defined, sharp tips and intact structures. Specifically, each patch covered an area of 0.56 cm^2^and consisted of 144 microneedles. Quantitative analysis revealed that the needles had a height of 500 μm, a base diameter of 300 μm, and an inter-needle spacing of 500 μm. SEM images further confirmed the formation of well-aligned conical needles with smooth surfaces and no observable defects (Fig. 1B1–B2). Bilayer compartmentalization was visualized using sodium fluorescein in the needle layer and rhodamine B in the PVA backing layer. Fluorescence imaging revealed clear spatial separation, with sodium fluorescein localized in the needle tips and rhodamine B confined to the backing layer, indicating minimal interlayer diffusion during fabrication (Fig. 1C-D).

The skin penetration capability of Scop-DMN was evaluated using in vitro insertion models. H&E staining of mouse dorsal skin following DMN application revealed well-defined microchannels penetrating the stratum corneum (Fig. 1E). Consistently, trypan blue staining of ex vivo porcine skin showed a dense array of uniformly distributed pores, with a complete skin insertion percentage of 92%, confirming effective skin penetration and microchannel formation (Fig. 1F). The force-displacement profile (Fig. 1H) exhibited no evidence of deformation or fracture within the tested range, indicating adequate mechanical strength for skin insertion. The profile demonstrates a continuous increase in force without the characteristic sudden “drop-off” that would indicate needle fracture or catastrophic structural failure within a displacement of 0.5 mm. These results confirm that the Scop-DMNs possess sufficient mechanical strength to penetrate the stratum corneum without premature breakage. The in vitro dissolution performance of Scop-DMN was further assessed using ex vivo porcine skin. After a total application time of 2 min, the DMN dissolved rapidly (Fig. 1G), with residual height less than 10% of the original height, indicating efficient and rapid drug release from the DMN system.

Method validation demonstrated good linearity over the concentration range of 0.3–5.0 μg/mL (r^2^ = 0.9993; regression equation: y = 19.542x + 184.07). The precision met the acceptance criteria, with intra-and inter-day RSD values ≤ 5%. Accuracy was confirmed by recovery rates ranging from 98.4% to 101.7%. The LOD (S/N = 3) and LOQ (S/*N* = 10) were determined to be 200 ng/mL and 300 ng/mL, respectively. No interference was observed from either the ultrapure water solvent or the blank microneedle matrix at the retention time of scopolamine hydrobromide, indicating satisfactory specificity of the method. The validated HPLC method exhibited good linearity, specificity, accuracy, and precision. It is simple, rapid, and reproducible, making it suitable for subsequent formulation evaluation and for providing a reference in the establishment of quality standards. Furthermore, We prepared three batches of Scop-DMN patches and determined their drug content to be 723.30 ± 38.11 ng each patch using the HPLC method. With a relative standard deviation of 5.27%, these results demonstrated that the drug-loaded DMN fabricated using this method exhibited excellent uniformity of drug content and batch-to batch stability. Moreover, the actual drug loading efficiency at the microneedle tip was 2.15%.

The transdermal delivery results of Scop-DMN and Scop-patch (without needles) showed that the residual drug amount in the DMN group fell below the limit of detection (200 ng/mL), whereas the residual rate in the patch (without needles) group remained at 73.28%, after 6 h of continuous application (Fig. 1I). Even after 12 h, over 30% of Scop remained on the skin surface for the patch group, indicating that the passive diffusion mechanism characteristic of traditional topical patches entails a significant delivery lag, rendering them unsuitable for rapid antidepressant therapy. In contrast, DMN delivery effectively bypasses the stratum corneum barrier, enabling rapid intradermal drug delivery, enhancing bioavailability, and facilitating a rapid antidepressant effect.

### 3.2 In vivo and in vitro biocompatibility evaluations

**Fig. 2:**
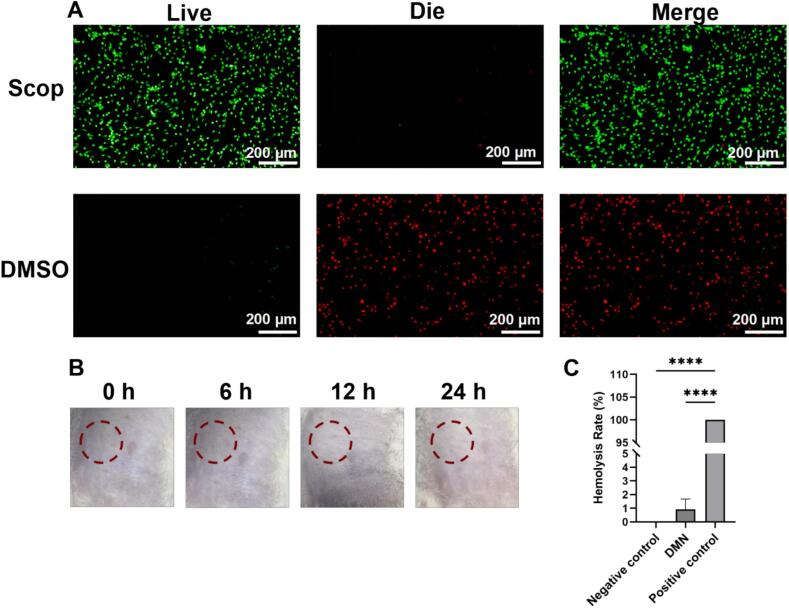
In vitro biocompatibility of Scop-DMN. (A) Dead/live staining of NIH/3 T3 cells (scale bars = 200 μm); (B) Skin irritation response on rat abdominal skin; (C) Erythrocyte hemolysis test data (*n* = 3).

The in vitro biocompatibility of Scop-DMN was evaluated by live/dead staining in fibroblasts and a hemolysis assay. In the live/dead assay, cells treated with Scop-DMN extracts displayed predominantly green fluorescence with only minimal red-positive cells, showing no significant difference compared with the negative control (Fig. 2A). In contrast, the DMSO-treated positive control exhibited intense red fluorescence and markedly reduced green signal, indicating severe membrane damage and cell death, thereby confirming the favorable cytocompatibility of the Scop-DMN formulation. Hemocompatibility was assessed by visual inspection of the supernatant after incubation with erythrocytes and by quantifying hemolysis rates (Fig. 2C). The negative control (PBS) remained clear, whereas the positive control (deionized water) exhibited a bright red supernatant, indicating complete erythrocyte lysis. In contrast, the Scop-DMN group closely resembled the PBS control, with no observable hemoglobin release. Consistently, the hemolysis rate of Scop-DMN was approximately 0%, well below the ISO 10993-4 threshold for non-hemolytic materials (<5%), confirming excellent hemocompatibility with negligible hemolytic activity.

Mouse skin irritation studies demonstrated that after continuous application of the DMN patch for 12 h, slight swelling was observed at the administration site (Fig. 2B). Importantly, the skin returned to its normal state within 12 h post-removal, with no observable signs of irritation such as edema or erythema. The mice exhibited normal feeding and activity patterns without scratching behavior. By 24 h, new hair growth was evident at the application site, indistinguishable from the surrounding skin, confirming the excellent biocompatibility of the Scop-DMN patch.

### 3.3 Behavioral testing

**Fig. 3:**
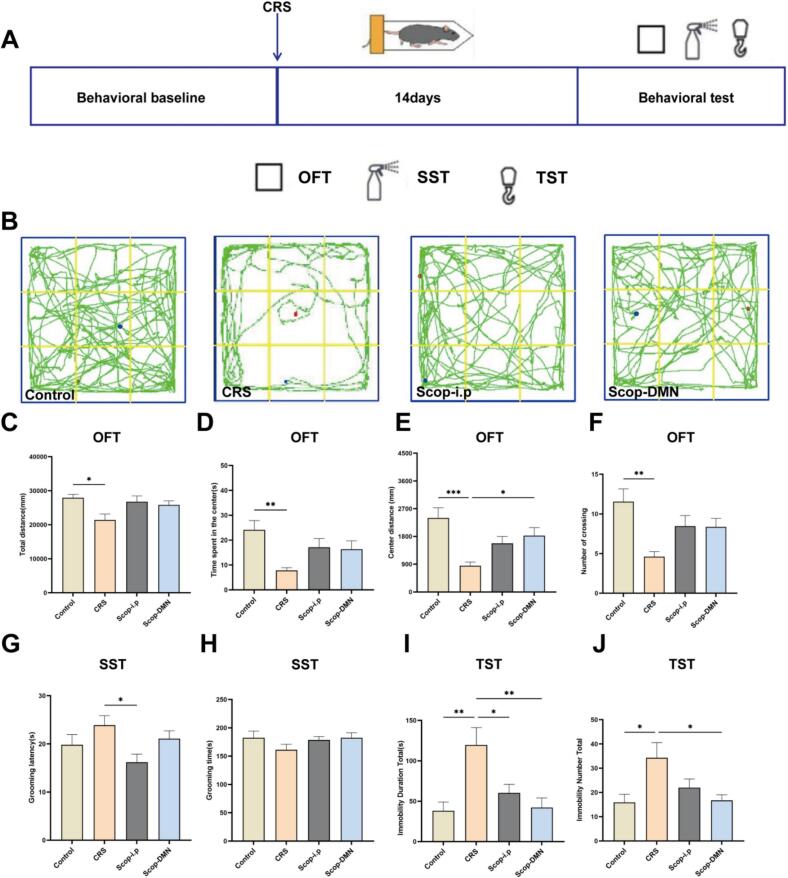
Scopolamine-loaded bilayer dissolving microneedles ameliorate depression-like behaviors in a chronic restraint stress (CRS) mouse model. (A) Schematic diagram of the experimental design. (B) Representative movement trajectories from the open field test (OFT). (C–F) Quantification of OFT parameters: total distance traveled (C), time spent in the center (D), distance traveled in the center (E), number of center crossings (F). (G-H) Quantification of sucrose splash test (SST) parameters: latency to groom (G) total grooming time (H). (I–J) Quantification of tail suspension test (TST) parameters: total immobility duration (I) number of immobility episodes (J). Data are presented as mean ± SEM. *P < 0.05, **P < 0.01, ***P < 0.001 (one-way ANOVA followed by Tukey's post hoc test).

Fig. 3A provides a schematic overview of the experimental timeline.

Locomotor activity and anxiety-like behaviors were assessed in the open field test (OFT). Representative trajectory plots illustrate that mice subjected to chronic restraint stress (CRS) displayed pronounced hypoactivity and avoidance of the central zone compared to unstressed Control mice (Fig. 3B). Quantitative analysis confirmed that the CRS group exhibited significant reductions in total distance traveled, number of entries into the central zone, distance traveled within the central zone, and time spent in the central zone (Fig. 3C-F). These deficits validate the successful establishment of a depression-like phenotype characterized by psychomotor retardation and thigmotaxis.

Previous studies have shown that microneedle-based drug delivery can improve bioavailability and therapeutic efficacy (Moawad et al., 2025). To assess this, we compared the antidepressant effects of scopolamine delivered via our bilayer dissolving microneedles (Scop-DMN) against conventional intraperitoneal (i.p.) injection. Relative to the CRS group, both the Scop-DMN and Scop-i.p treatments produced a robust reversal of the behavioral deficits, significantly increasing total distance, central entries, central distance, and central time. Notably, the Scop-DMN group demonstrated significantly greater efficacy than the Scop-i.p group across all measured parameters, indicating that transdermal delivery via Scop-DMNs elicits a rapid and superior antidepressant-like effect compared with conventional systemic administration.

To evaluate anhedonia and self-care behavior, the sucrose splash test (SST) was performed. As shown in Fig. 3G, mice in the CRS group exhibited a significantly longer latency to initiate grooming compared to the Control group, reflecting a deficit in motivated behavior. Both Scop-DMN and Scop-i.p administration reversed this effect, reducing grooming latency to levels comparable with the Control group. No significant differences in the total duration of grooming were observed among any of the experimental groups (Fig. 3H).

Depressive-like behavioral despair was quantified using the tail suspension test (TST). The CRS group exhibited a significant increase in both the total duration of immobility and the number of immobility episodes compared to the Control group, confirming a robust despair-like phenotype (Fig. 3I-J).

Both Scop-DMN and Scop-i.p treatments significantly attenuated these behaviors. Scop-DMNs reduced both the total immobility duration and the number of immobility episodes. In contrast, while i.p. scopolamine reduced immobility duration, it did not significantly alter the number of immobility episodes. Critically, the Scop-DMN group demonstrated superior efficacy, showing a greater reduction in both total immobility duration and the number of immobility episodes compared to the i.p group. These results indicate that DMN-mediated delivery of scopolamine produces a more potent and comprehensive antidepressant-like effect than conventional systemic administration.

### 3.4 Scopolamine-loaded bilayer dissolving microneedles rescue synaptic protein deficits in the prefrontal cortex via BDNF/mTOR pathway activation

Extant literature indicates that in murine models of depression, protein expression within the BDNF/mTOR signaling pathway is significantly downregulated in the mPFC, a dysregulation that can be reversed by subtherapeutic doses of scopolamine (Kan et al., 2022; Liu et al., 2021). To elucidate the molecular mechanism underlying the antidepressant-like effects of Scop-DMNs, we quantified the expression of BDNF, P-mTOR, PSD95, and SynI in the mPFC 24 h post-administration.

**Fig. 4:**
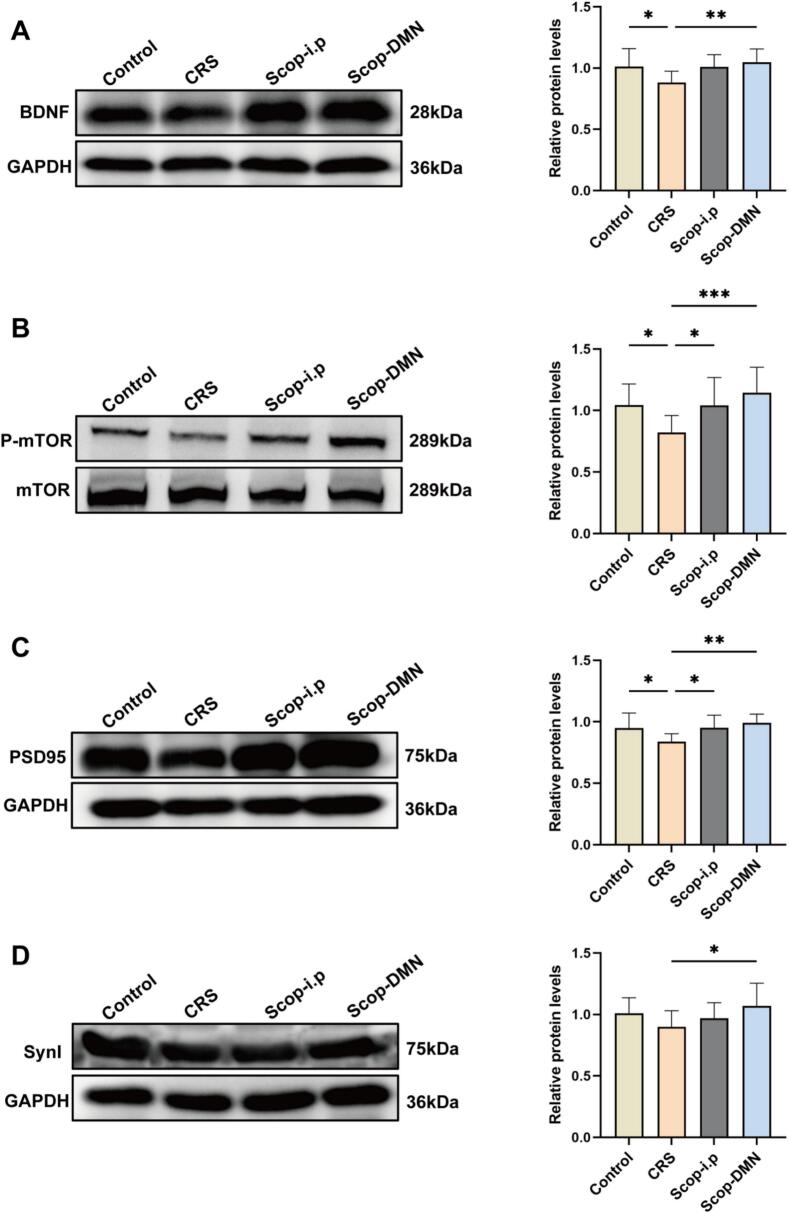
Scopolamine-loaded bilayer dissolving microneedles (DMNs) restore synaptic plasticity-associated protein expression in the mPFC of CRS-induced depressed mice.(A) Representative immunoblots (left) and densitometric quantification (right) of BDNF protein levels. (B—D) Representative immunoblots and quantitative analysis of P-mTOR/mTOR, PSD95, and SynI, respectively. GAPDH or total mTOR were used as internal loading controls for normalization. Data are presented as mean ± SEM. *P < 0.05, **P < 0.01, ***P < 0.001 (one-way ANOVA followed by Tukey's post hoc test).

Western blot analysis revealed that chronic restraint stress (CRS) markedly impaired neurotrophic signaling and synaptic integrity. Compared with the Control group, the CRS model showed significant reductions in BDNF, P-mTOR, and PSD95 (*P < 0.05), while SynI displayed a decreasing trend that did not reach statistical significance (Fig. 4A-D).

Both scopolamine interventions alleviated these CRS-induced molecular deficits, with Scop-DMN exhibiting a more robust restorative profile. Specifically, Scop-DMN significantly increased BDNF relative to the CRS group (*P < 0.05 vs. CRS) and restored it toward near-basal levels, whereas i.p. administration did not significantly alter BDNF (*P* > 0.05 vs. CRS) (Fig. 4A). For mTOR pathway activation, Scop-DMN upregulated P-mTOR more strongly than i.p. dosing (***P < 0.001 vs. CRS; **P < 0.01 vs. i.p.), while the i.p. group produced only a moderate increase (Fig. 4B). Consistently, Scop-DMN also induced a greater recovery of downstream synaptic markers, significantly elevating PSD95 (**P < 0.01 vs. CRS; *P < 0.05 vs. i.p.) and SynI (*P < 0.05 vs. CRS) (Fig. 4C–D). In contrast, i.p. scopolamine led to partial restoration of P-mTOR and PSD95 but failed to significantly affect SynI (P > 0.05 vs. CRS).

Taken together, these data indicate that transdermal delivery of scopolamine via dissolving microneedles more effectively reactivates the BDNF/mTOR axis and promotes a more coordinated normalization of synaptic protein expression in the mPFC, providing a molecular basis for the superior behavioral outcomes observed with Scop-DMN treatment.

## 4 Discussion

This study addresses a critical bottleneck in scopolamine-based rapid antidepressant therapy: the paradox between the necessity for rapid cerebral delivery and the risk of systemic anticholinergic side effects.While conventional transdermal scopolamine patches are clinically available, their application is predominantly restricted to the prevention of motion sickness (Atif et al., 2022; Navarria et al., 2015). These traditional patches utilize sustained-release technology dependent on passive diffusion across the stratum corneum. Consequently, they exhibit a significantly delayed onset of action, requiring application 4 to 6 h prior to exposure to effectively desensitize the vestibular nerve and inhibit gastrointestinal peristalsis. However, the primary objective of this study is to harness scopolamine for a rapid antidepressant effect. Achieving rapid-acting antidepressant efficacy necessitates a swift systemic absorption profile and immediate CNS penetration, which conventional slow-release patches fail to provide.

Unlike conventional transdermal preparations, microneedles successfully pierce the formidable stratum corneum barrier, delivering the therapeutic payload directly into the dermal microvascular network. This mechanism facilitates the rapid entry of scopolamine into the systemic circulation and subsequently the CNS, effectively accelerating the onset of the antidepressant response. By replacing traditional administration routes with a microneedle dosage form, we offer a highly operable, minimally invasive, and self-administrable strategy that is ideally suited for the rapid management of depressive symptoms.

The structural innovation of the bilayer design (drug-loaded tip layer and a PVA backing layer) is pivotal to its performance. The mechanical robustness of the fabricated Scop-DMN patch was evaluated by analyzing the force-displacement relationship during skin insertion. The patch, comprising 144 microneedles, sustains an average applied force of approximately 0.076 N per microneedle at a displacement of 0.5 mm. This value significantly exceeds the 0.058 N per microneedle required for effective skin penetration (Chew et al., 2020). Consequently, the Scop-DMN patch exhibits superior mechanical strength, ensuring reliable skin insertion and confirming its suitability for practical clinical applications. Compared to integrated microneedles, tip-loaded microneedles can significantly improve drug utilization and ensure the accuracy and controllability of transdermal drug delivery dosage. Furthermore, this design eliminates the risk of needle residue and enhances operational safety, facilitating a painless and autonomous self-administration scenario that is highly desirable for patients with depression. Similar therapeutic advantages of layered (multicompartment) microneedle platforms have also been demonstrated in other disease settings, where spatiotemporal control over cargo release enables robust efficacy while maintaining a minimally invasive, patient-friendly administration. For example, “Multicompartmental microneedle arrays for transdermal delivery of multiple vaccines” and “Dissolving microneedle patch for transdermal delivery of insulin” reported that layered/dissolving microneedle designs achieved efficient delivery and strong pharmacological/immunological responses, supporting the broader translational potential of stratified microneedle architectures for diverse therapeutic indications(Chen et al., 2018; Halder et al., 2021).

The behavioral results underscore the superior efficacy of Scop-DMNs over conventional i.p. administration across multiple dimensions of depressive-like behavior. Scop-DMN treatment led to more robust improvements in exploratory activity in the open field test (OFT), a more pronounced recovery of self-care motivation in the sucrose splash test (SST), and a significant reduction in despair behavior during the tail suspension test (TST). Notably, the enhanced behavioral benefit of Scop-DMNs may be attributable, at least in part, to pharmacokinetic and route-dependent advantages of transdermal/intradermal delivery. First, whereas i.p. dosing can result in substantial presystemic elimination due to portal drainage and hepatic first-pass metabolism, microneedle-enabled intradermal administration is generally considered to bypass gastrointestinal processing and reduce first-pass loss, thereby potentially increasing systemic bioavailability and improving exposure at an equivalent nominal dose(Kushwaha et al., 2026; Naik et al., 2000). Second, although dissolving microneedle tips dissolve rapidly, drug deposited within the skin can plausibly form an intradermal “depot” that releases over time through dermal microcirculation and lymphatic uptake, which may stabilize systemic—and consequently central—drug levels and help explain the more persistent behavioral improvements observed at the 24 h time point relative to i.p. injection(Moawad et al., 2025; Yang et al., 2021).

Mechanistically, scopolamine’ s rapid antidepressant action has been widely linked to activation of the BDNF/mTOR signaling axis in the medial prefrontal cortex (mPFC), which promotes synaptic protein synthesis and restores stress-impaired synaptic plasticity. In the prevailing model proposed by Duman and others, muscarinic receptor blockade on inhibitory interneurons transiently disinhibits pyramidal neurons, triggering a glutamatergic burst and AMPAR-dependent postsynaptic activation. This initiates activity-dependent BDNF release and subsequent mTOR engagement, thereby enhancing the translation of synaptic proteins (e.g., PSD95 and SynI), accelerating synaptogenesis, and strengthening mPFC connectivity to counteract stress-induced synaptic deficits(Ghosal et al., 2018; Navarria et al., 2015).

Consistent with this framework, our western blot results demonstrate that CRS produced a measurable suppression of this plasticity-related cascade, reflected by reduced BDNF and decreased mTOR pathway activation (lower P-mTOR), accompanied by downregulation of synaptic markers (PSD95 and SynI). Importantly, both scopolamine delivery routes showed a corrective trend, but Scop-DMN produced a more complete molecular rescue across the pathway. Specifically, Scop-DMN significantly increased BDNF relative to the CRS group and restored it toward control levels, whereas i.p. scopolamine did not produce a comparably robust normalization of BDNF in our dataset. At the signaling level, Scop-DMN elevated P-mTOR more strongly than i.p. administration and showed clearer recovery from CRS-induced suppression, indicating more effective engagement of mTOR-dependent translational control. In parallel, Scop-DMN produced a more pronounced restoration of PSD95 compared with i.p. dosing, consistent with improved postsynaptic structural recovery. For SynI, Scop-DMN again showed a significant increase versus i.p administration, suggesting better normalization of presynaptic functional capacity.

## 5 Conclusion

In summary, we have successfully developed a novel bilayer dissolving microneedle system for the transdermal delivery of scopolamine, providing an innovative solution for the rapid treatment of depression. The Scop-DMNs demonstrated superior antidepressant-like efficacy compared to systemic i.p. administration, characterized by enhanced behavioral recovery and more potent activation of the BDNF/mTOR synaptic repair pathway in the mPFC. By integrating formulation innovation with mechanistic validation, this study establishes a “precise delivery–rapid brain entry–synaptic repair” therapeutic chain. Our findings suggest that Scop-DMNs represent a promising, minimally invasive, and self-administrable platform for the management of acute depression, offering a significant advancement in neuropharmacological delivery strategies.

## CRediT authorship contribution statement

**Yanping Wang:** Writing – original draft, Investigation, Formal analysis. **Yuwei Shi:** Writing – original draft, Investigation, Formal analysis. **Qiuyue Chen:** Writing – review & editing, Data curation. **Minghui Hu:** Writing – review & editing, Investigation. **Yue Zhao:** Writing – review & editing, Data curation. **Wanqing Ren:** Writing – review & editing, Investigation. **Kun Liu:** Writing – review & editing, Investigation. **Fangyi Hou:** Writing – review & editing, Investigation. **Yaru Cui:** Writing – review & editing, Investigation. **Yuning Ma:** Writing – review & editing, Supervision, Methodology. **Mengzhen Xing:** Writing – review & editing, Supervision, Resources, Methodology. **Xiwen Geng:** Writing – review & editing, Supervision, Resources, Project administration, Funding acquisition, Conceptualization.

## Declaration of competing interest

The authors declare that they have no known competing financial interests or personal relationships that could have appeared to influence the work reported in this paper.

## Data Availability

Data will be made available on request.
